# Expression of SART3 antigen and induction of CTLs by SART3-derived peptides in breast cancer patients

**DOI:** 10.1054/bjoc.2000.1690

**Published:** 2001-04

**Authors:** Y Suefuji, T Sasatomi, S Shichijo, S Nakagawa, H Deguchi, T Koga, T Kameyama, K Itoh

**Affiliations:** Departments of Oral and Maxillofacial Surgery; Surgery; Immunology, Kurume University School of Medicine, Kurume, Fukuoka, Japan

**Keywords:** SART3 antigen, breast cancer, HLA-A24, immunotherapy, cancer vaccine

## Abstract

We recently reported the SART3 tumour-rejection antigen as possessing tumour epitopes capable of inducing HLA-class I-restricted cytotoxic T lymphocytes (CTLs). This study investigated expression of the SART3 antigen in breast cancer to explore an appropriate molecule for use in specific immunotherapy of breast cancer patients. The SART3 antigen was detected in all of the breast cancer cell lines tested, 30 of 40 (75%) breast cancer tissue samples, and 0 of 3 non-tumourous breast tissue samples. SART3 derived peptides at positions 109–118 and 315–323 induced HLA-A24 restricted CTLs that reacted to breast cancer cells from the peripheral blood mononuclear cells (PBMCs) of breast cancer patients. Therefore, the SART3 antigen and its peptides could be an appropriate molecule for use in specific immunotherapy of the majority of HLA-A24-positive breast cancer patients. © 2001 Cancer Research Campaignhttp://www.bjcancer.com


British Journal of Cancer (2001) **84**(7), 915–919
© 2001 Cancer Research Campaign
doi: 10.1054/ bjoc.2000.1690, available online at http://www.idealibrary.com on IDEAL®

http://www.bjcancer.com

### Expression of SART3 antigen and induction of CTLs by SART3-derived peptides in breast cancer patients

**Y Suefuji[1], T Sasatomi[2], S Shichijo[3], S Nakagawa[2], H Deguchi[2], T Koga[2], T Kameyama[1] and K Itoh[3]**

Departments of [1]Oral and Maxillofacial Surgery, [2]Surgery, and [3]Immunology, Kurume University School of Medicine, Kurume, Fukuoka, Japan

**Summary** We recently reported the SART3 tumour-rejection antigen as possessing tumour epitopes capable of inducing HLA-class I-restricted cytotoxic T lymphocytes (CTLs). This study investigated expression of the SART3 antigen in breast cancer to explore an appropriate molecule for use in specific immunotherapy of breast cancer patients. The SART3 antigen was detected in all of the breast cancer cell lines tested, 30 of 40 (75%) breast cancer tissue samples, and 0 of 3 non-tumourous breast tissue samples. SART3 derived peptides at positions 109–118 and 315–323 induced HLA-A24 restricted CTLs that reacted to breast cancer cells from the peripheral blood mononuclear cells (PBMCs) of breast cancer patients. Therefore, the SART3 antigen and its peptides could be an appropriate molecule for use in specific immunotherapy of the majority of HLA-A24-positive breast cancer patients. © 2001 Cancer Research Campaign http://www.bjcancer.com

**Keywords**: SART3 antigen; breast cancer; HLA-A24; immunotherapy; cancer vaccine

The incidence of breast cancer has been increasing since the 1960s at the global level. The 5-year survival rate is generally low in stage III breast cancer patients, and extremely low in the stage IV patients, regardless of different treatment modalities including hormone therapy, chemotherapy and radiotherapy. Therefore, development of new treatment modalities is needed, and one of them will be specific immunotherapy (Nestle et al, 1998; Rosenberg et al, 1998; Marchant et al, 1999). However, little information is available regarding tumour-rejection antigens on breast cancers. Many tumour-rejection antigens have been identified from melanomas (van der Bruggen et al, 1991, 1994; Traversari et al, 1992; Kawakami et al, 1994, 1995; Robbins et al, 1996, 1997), but these antigens were rarely expressed in breast cancers (Vincenzo et al, 1995). $SART1_{259}$ antigen that was identified from an oesophageal cancer was also rarely expressed in breast cancers (Kawamoto et al, 1999). MUC1 antigen and HER2/neu oncogene products might encode a tumour epitope on HLA-$A2^{+}$ breast cancers (David et al, 1995; Reddish et al, 1998), but the peptide specificity and HLA-restriction have not been clearly demonstrated.

We recently reported that the SART3 tumour-rejection antigen possesses tumour epitopes capable of inducing HLA-A24-restricted CTLs in head and neck cancer patients (Yang et al, 1999). The 140 kD of the SART3 antigen was expressed in the nucleus of all of the malignant cell lines and in the majority of cancer tissues tested, but not in the nucleus of any of the normal tissues except that of the testis. It was also expressed in the cytosol of the majority of proliferating cells, including normal cells and malignant cells.

In this study, we investigated expression of the SART3 antigen in breast cancer tissues to explore for an appropriate molecule useful for specific immunotherapy of breast cancer, and found evidence suggesting that the SART3 could be an appropriate target molecule.

*Received 25 July 2000*
*Revised 21 December 2000*
*Accepted 21 December 2000*

*Correspondence to:* K Itoh: Email: Kyogo@med.kurume-u.ac.jp

### MATERIALS AND METHODS

### Samples

The eight breast cancer cell lines used in this study were MCF7, R27, OCUB-M, OCUB-F, YMB-1-E, CRL1500, MDA-MB-231 and T-47D. These cell lines were incubated with RPMI 1640 medium, EME medium, or Dulbecco's Modified Eagle's Medium supplemented with 10% FCS, included in these cell lines reported (Kawamoto et al, 1999). The HLA-$A2402^{+}$ tumours are OCUB-M and OCUB-F.

Breast cancer tissue samples ($n$ = 40) and non-tumourous breast tissue samples ($n$ = 3) were obtained at the time of surgery in the Kurume University Hospital. All of the cancers were histologically determined to be adenocarcinoma. In samples, stage I and stage II group numbered 23 cases and stage III and stage IV numbered 17 cases. A section of each sample was minced with scissors and kept at –80˚C until use. The KE -4 oesophageal squamous carcinoma cell line (HLA-A2402/A2601) from which the SART3 was cloned (Yang et al, 1999) was used as a positive control. VA13 fibroblast cells (no expression of HLA-class I alleles), and CIR-A402 cells were used as target cells as negative controls, as reported previously (Yang et al, 1999; Murayama et al. 2000).

### Western blot analysis

Expression of the SART3 antigen in samples was investigated by Western blot analysis using the polyclonal anti-SART3 antibody as reported previously (Yang et al, 1999).

### Expression of oestrogen and progesterone receptors

Expression of the oestrogen or progesterone receptors in frozen sections was measured using the standard enzyme immunoassay commercially available from SRL (Tokyo). For the assays Abbot

ER-EIA mAb or Abbott PgR-EIA mAb (Abbott Park) was used according to the manufacturer's instructions. The international UICC-TNM system was used for staging the 31 breast cancer patients.

### Peptides and CTL induction

The $SART3_{109–118}$ (VYDYNCHVDL) and $SART3_{315–323}$ (AYIDFEMKI) peptides were used for CTL induction as described previously (Yang et al, 1999). PBMCs from breast cancer patients ($n$ = 4) were incubated with 10 μM of a peptide in one well of a 24-well plate containing 2 ml of culture medium with 100 units $ml^{-1}$ of IL-2 (Shiongi Pharm Co, Osaka). At days 7, 14 and 21 of culture, the cells were harvested, washed and re-incubated with the irradiated (45 Gy) autologous PBMCs acting as antigen-presenting cells, which had been pre-incubated with the same peptide at the same dose for 3 h. These peptide-stimulated PBMCs ($2–3 \times 10^3$ cells $well^{-1}$) were harvested at day 28 of culture, and further cultured in a 96-well U-bottom microculture plate in the presence of feeder cells (irradiated HLA-$A24^+$ PBMCs) that had been pre-pulsed with a corresponding peptide in order to obtain large numbers of effector cells. Seven to 10 days later, the expanded cells were transferred to wells of a 24-well plate and were incubated in the absence of either a peptide or feeder cells for an additional 21–28 days.

The surface phenotypes and CTL activity of these cells was tested by an IFN-γ production assay (limit of sensitivity = 10 pg $ml^{-1}$), and $^{51}$Cr-labelled assays at different E:T ratios at the triplicate assays. For preparation of CTL sublines, the peptide-stimulated PBMCs were incubated at 1 or 10 or 100 cells per well in the presence of irradiated HLA-A24 PBMCs ($2–3 \times 10^5$ cells $well^{-1}$) that were pre-loaded with a relevant peptide, followed by testing of their surface phenotype and CTL activity to produce IFN-γ in response to CIR-A2402 pre-loaded with a peptide.

The surface phenotype of effector cells was investigated by an immunofluorescence assay with FITC-conjugated anti-CD3, -CD4 or -CD8 mAb (Yang et al, 1999). For inhibition of CTL activity, 20 mg $ml^{-1}$ of anti-class I (W6/32, IgG2a) or anti-CD8 (IgG2a), anti-class II (H-DR-1, IgG2a) and anti-CD4 (IgG1) mAb were used as reported previously. Anti-CD13 (MCS-2, IgG1) and anti-CD14 (H14, IgG2a) mAb were used as isotype-matched control mAb.

### RESULTS

### Expression of SART3 antigen

The 140 kD of the SART3 antigen was expressed in both the cytosol and nuclear fractions of all eight breast cancer cell lines tested. This band was also detectable in the cytosol fraction of 28 of 40 (70%) breast cancer tissue samples, and in the nuclear fraction of 30 of 40 (75%) breast cancer tissue samples. The bands other than the 140 kD, which were observed in cancer samples, were evaluated as non-specific since the polyclonal antibody was used for the detection.

The expression of SART3 antigen in the group of stage I and II was 18 of 23 samples (78.3%) in the cytosol, and 19 of 23 samples (82.6%) in the nucleus, while that in the group of the stage III and IV was 10 of 17 samples (58.8%) in the cytosol, and 11 of 17 samples (64.7%) in the nucleus. However, it was not detectable in either the cytosol or nuclear fraction of any of the normal breast tissue samples tested. Examples of results are shown in Figure 1, and a summary of these results is presented in Table 1.

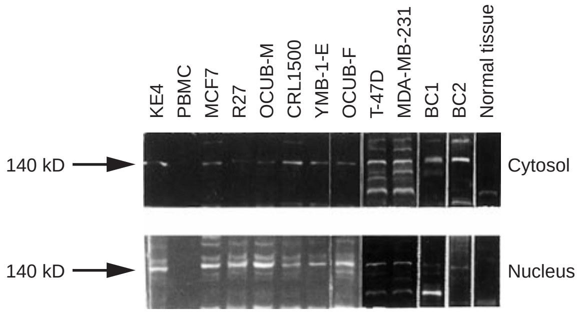

**Figure 1** Expression of the SART3 antigen in the breast cancer cell lines, breast cancer tissue samples, and non- tumourous breast tissue samples was investigated by Western blot analysis with polyclonal anti-SART3 antibody (Yang et al, 1999). Representative results of the investigation of both cytosol and nuclear fractions are shown. PBMCs of a healthy donor were used as a negative control and KE4 oesophageal cancer cells as a positive control. Breast cancer cell lines included MCF7, R27, OCUB-M, CRL1500, YMB-1-E, OCUB-F, T-47D, and MDA-MB-231. Breast cancer tissues are shown as BC1 and BC2 in the figure, while non-tumour breast tissue is shown as normal tissue.

### Expression of oestrogen or progesterone receptors

The expression of oestrogen receptor (ER) or progesterone receptor (PR) in these tumours was investigated in view of potential combined immunotherapy and hormone therapy. Twenty-three and 17 of the 31 breast cancer tumours expressed ER and PR, respectively (Table 1). Among the 31 tumours, 16 tumours were $SART3^+$ and $ER^+$, two tumours were $SART3^+$ and $ER^-$, seven tumours were $SART3^-$ and $ER^+$. The remaining six tumours were $SART3^-$ and $ER^-$. Similarly, 11 tumours were $SART3^+$ and $PR^+$, seven tumours were $SART3^+$ and $PR^-$, six tumours were $SART3^-$ and $PR^+$, and seven tumours were $SART3^-$ and $PR^-$.

**Table 1** Expression of SART3 antigen in breast cancer

| Samples | | Numbers | SART3 antigen expression | | Oestrogen receptor | Progesterone receptor |
|---|---|---|---|---|---|---|
| | | | Cytosol | Nucleus | | |
| Breast cancer cell lines | | 8 | 8/8 | 8/8 | ND | ND |
| | Stage I, II | 23 | 18/23 (78.3%) | 19/23 (82.6%) | 16/19 (84.2%) | 13/19 (68.4%) |
| Breast cancer tissues | Stage III, IV | 17 | 10/17(58.8%) | 11/17 (64.7%) | 8/12 (66.7%) | 4/12 (33.3%) |
| | Total | 40 | 28/40 (70.0%) | 30/40 (75.0%) | 23/31(74.2%) | 17/31(54.8%) |
| Non-tumourous breast tissues | | 3 | 0/3 | 0/3 | ND | ND |

ND = not determined; percentages of positive

 *© 2001 Cancer Research Campaign*

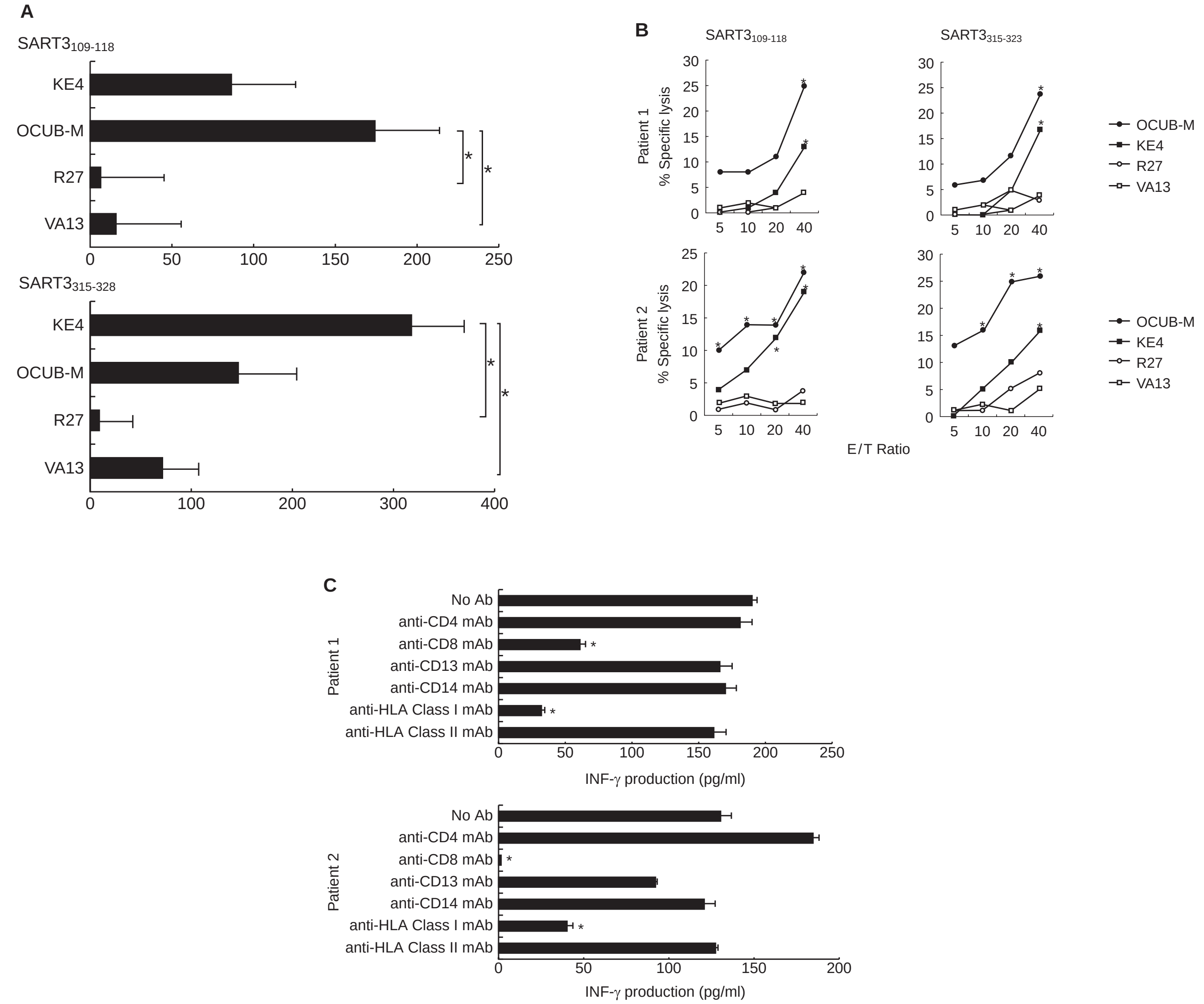

**Figure 2** CTL activity of the peptide-induced PBMCs. PBMCs stimulated with $SART3_{109–118}$ or $SART3_{315–323}$ peptide were tested for their ability to recognize breast cancer cells by IFN-γ assay (**A**) and 6-h $^{51}$Cr release assay (**B**). (**A**) Values represent the mean of IFN-γ produced from the peptide-stimulated PBMCs of four breast cancer patients. Background IFN-γ production by the PBMCs alone (50–100 pg $ml^{-1}$) has been subtracted. (**B**) Values represent the mean of % specific lysis of the triplicate determinants of the peptide-stimulated PBMCs of patients 1 and 2. Breast cancer cells used were OCUB-M ($HLA-A2402^+$), and R27 ($HLA-A2402^–$). The KE4 tumour cell line was used as a positive control, while VA13 cell line was a negative control. (**C**) These PBMCs showing HLA-24-restricted CTL activity were tested for their ability to produce IFN-γ in response to $HLA-24^+$ breast cancer cells (OCUB-M) at an E:T ratio of 2 in the presence of 20 mg $ml^{-1}$ of anti-CD4 (Nu-TH/1), anti-CD8 (Nu-TS/C), anti-CD13 (MCS-2), anti-CD14 (H14), anti-class 1 (W6/32), or anti-class II (DR) mAb. Values represent the mean of duplicate assays. The two-tailed Student's *t*-test was employed for statistical analysis ($*P \leq 0.05$)

### Induction of CTLs by the SART3 peptides

The $SART3_{109–118}$ and $SART3_{315–323}$ peptides were tested for their ability to induce CTLs from the PBMCs of $HLA-A24^+$ patients with breast cancer ($n$ = 4, histologically-determined adenocarcinoma). These PBMCs produced a significant level of IFN-γ by recognition of $HLA-A24^+$ cancer cells (KE-4 and OCUB-M) when stimulated in vitro with either $SART3_{109–118}$ or $SART3_{315–323}$ peptide (Figure 2A). In contrast, they failed to react to $HLA-A24^–$ breast cancer cells (R27). These effector cells consisted of 20–30% $CD3^+CD4^-CD8^+$ T cells and 70–80% $CD3^+CD4^+CD8^-$ T cells (data not shown). The HLA-A24-restricted CTL activity in these PBMCs was confirmed by a 6-h $^{51}$Cr release assay after further expansion in patients 1 and 2. These peptide-stimulated PBMCs showed significant levels of cytotoxicity against the $HLA-A24^+$ tumour cells (KE-4 and OCUB-M), but not against $HLA-A24^–$ tumour cells (VA13 and R27) (Figure 2B).

The CTL activity was inhibited by anti-class I (W6/32) or anti-CD8 (Nu-TS/C), but not by anti-CD4 (Nu-TH/I), anti-class II (DR), anti-CD13 (MCS-2) or anti-CD14 (H14) mAb, taken as a negative control (Figure 2C). Further, CTL sublines were established and tested for their reactivity to a peptide in order to confirm the peptide specificity in the CTLs.

One of six or three of 20 sublines from the PBMCs of patient 1, when stimulated with the $SART3_{109–118}$ peptide or $SART3_{315–323}$ peptide, produced a significant amount of IFN-γ by recognition of C1R-A2402 cells pulsed with a corresponding peptide, respectively (Figure 3A and B). Similarly, one of three or four of 20 sublines from PBMCs of patient 2, when stimulated with the $SART3_{109–118}$ peptide or $SART3_{315–323}$ peptide, produced a significant

© 2001 Cancer Research Campaign

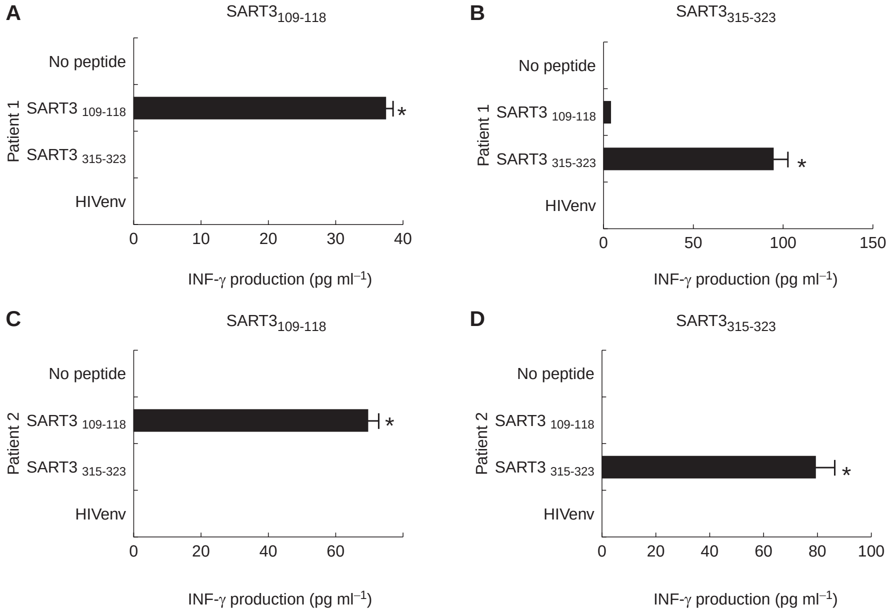

**Figure 3** Peptide specificity of CTL sublines was established by incubation of the PBMCs of patients 1 and 2, which had been stimulated with the $SART3_{109–118}$ or $SART3_{315–323}$ peptide. HLA-A2402-binding HIV peptide (RYLRDQQLLGI) was used as a negative control. One of six or three of 20 sublines from the PBMCs of patient 1, when stimulated with the $SART3_{109–118}$ peptide or $SART3_{315–323}$ peptide, produced a significant amount of IFN-γ by recognition of C1R-A2402 cells pulsed with a corresponding peptide, respectively (**A, B**). Similarly, one of three or four of 20 sublines from the PBMCs of patient 2, when stimulated with the $SART3_{109–118}$ peptide or $SART3_{315–323}$ peptide, produced a significant amount of IFN-γ by recognition of C1R-A2402 cells pulsed with a corresponding peptide, respectively (**C, D**). The other sublines failed to show peptide specificity. Values represent the mean of duplicate assays. The two-tailed Student's *t*-test was employed for statistical analysis (*$P \leq 0.05$)

amount of IFN-γ by recognition of C1R-A2402 cells pulsed with a corresponding peptide, respectively (Figure 3C, D).

### DISCUSSION

This study showed that the SART3 antigen encoding tumour epitopes capable of inducing CTLs was expressed in both the cytosol and nuclear fractions of all breast cell lines and the majority of breast tumours tested. In contrast, this antigen was undetectable in non-tumourous breast tissue samples. We recently obtained evidence that this is a RNA-binding protein involved in splicing regulation of RNA (Harada, unpublished results). These results suggest that the SART3 antigen plays an important role in cellular proliferation. This issue is now under investigation in our laboratory.

The present study also showed that the $SART3_{109–118}$ and $SART_{315–323}$ peptide induced HLA-A24-restricted CTLs recognizing the $SART3^+$ tumour cells including breast cancer cells in the PBMCs of all $HLA\text{-}A24^+$ breast cancer patients tested. These CTLs failed to lyse either $HLA\text{-}A24^-$ tumour cells (Figure 2) or $HLA\text{-}A24^+$ normal T cells (PHA-blastic cells) (data not shown). These results indicate the presence of CTL precursors reacting to the SART3 epitope on breast cancer cells in the circulation of the majority of breast cancer patients.

In contrast to the SART3 antigen, the other tumour antigens (MAGE-1, MAGE-4, $SARTI_{259}$, MUC1) were expressed in only a portion of breast cancers (Vincenzo et al, 1995; Reddish et al, 1998; Kawamoto et al, 1999). Further, no peptides were proven to have the ability to induce HLA-class I restricted CTLs reacting to breast cancer cells. Administration of HER2/neu peptide capable of binding to HLA-A2 molecules failed to induce the CTL activity against cancer cells (Zaks and Rosenberg, 1998). Therefore, the SART3 antigen and its peptides could be one of the most appropriate molecules for use in specific immunotherapy of breast cancer patients.

There are few treatment modalities available for patients with breast cancer which is lacking ER or resistant to the existing hormone therapy (Slamon et al, 1989; Teixeira et al, 1995; Yamauchi et al, 1997). Among 31 tumours tested, 16 (52%) expressed both SART3 and ER, two (7%) were $SART3^+$ and $ER^-$, seven (23%) were $SART3^-$ and $ER^+$, and six (19%) were $SART3^-$ and $ER^-$. These results suggest that a substantial proportion of breast cancer patients would be appropriate candidates for specific immunotherapy with the SART3 peptides, either administered independently or in combination with hormone therapy. Similar results were obtained with PR. Further, SART3 expression in the group of stage I and II was higher than that in the group of stage III and IV. A similar trend was observed in the expression of ER and PR. These results suggest that both immunotherapy and hormone therapy are more effective for breast cancer patients with early stage rather than the patients with advanced stage, although details involved in this phenomenon is not clear at the present time.

The HLA-A24 allele is found in 60% of Japanese, 20% of Caucasians and 12% of Africans (Imanishi et al, 1992). We have recently identified tumour-epitopes of the SART3 antigen on HLA-A0207 molecules, which are able to induce HLA-A2-restricted and tumour-specific CTLs in the PBMCs of epithelial cancer patients (Ito et al, unpublished results). The HLA-A2 allele is found in 40% of Japanese, 50% of Caucasians and 12% of Africans (Imanishi et al, 1992).

© 2001 Cancer Research Campaign

Subsequently, the SART3 antigen and its peptides could be an appropriate molecule for use in specific immunotherapy of HLA-A24[+] or -A2[+] breast cancer patients. These findings could provide important information for the development of a specific immunotherapy for a relatively large number of breast cancer patients all over the world.

### ACKNOWLEDGEMENTS

We thank Dr Kunzo Orita, an Executive Director of Hayashibara Biochemical Lab Inc, Japan, for providing the natural human IFN-γ with which to develop an IFN-γ ELISA system. This study was supported in part by Grants-in-Aid from the Ministry of Education, Science, Sports, and Culture of Japan (08266266), and from the Ministry of Health and Welfare, Japan (H10-genome-003).

### REFERENCES

David CL, Peter SG, George EP, Selwyn OR and Timothy JE (1995) Tumor-specific and HLA-A2-restricted cytolysis tumor-associated lymphocytes in human metastatic breast cancer. *J Immunol* **155**: 4486–4491

Gaugler B, van den Eynde B, van der Bruggen P, Romero P, Gaforio JJ, de Plaen E, Lethe B, Brasseur F and Boon T (1994) Human gene MAGE-3 codes for an antigen recognized on a melanoma by autologous cytolytic T lymphocytes. *J Exp Med* **179**: 921–930

Imanishi T, Akazawa T, Kimura A, Tokunaga K and Gojobori T (1992) Allele and haplotype frequencies for HLA and complement loci in various ethnic groups. In: *HLA 1991*, Vol. 1. Tsuji K, Aizawa M and Sasazuki (eds) pp 1065–1220. Oxford Scientific Publications, Oxford

Inoue H, Mori M, Honda M, Li J, Shibata K, Mimori K, Ueo H and Akiyoshi T (1995) The expression of tumor-rejection antigens of MAGE genes. *Int J Cancer* **62**: 523–526

Kawakami Y, Eliyahu S, Sakaguchi K, Robbins PF, Rivoltini L, Yannelli JR, Appella E and Rosenberg SA (1994) Identification of the immunodominant peptides of the MART-1 human melanoma antigen recognized by the majority of HLA-A2-restricted tumor infiltrating lymphocytes. *J Exp Med* **180**: 347–352

Kawakami Y, Eliyahu S, Jennings C, Sakaguchi K, Kang X, Southwood S, Robbins PF, Sette A, Appella E and Rosenberg SA (1995) Recognition of multiple epitopes in the human melanoma antigen gp100 by tumor-infiltrating T lymphocytes associated with in vivo tumor regression. *J Immunol* **154**: 3961–3968

Kawamoto M, Shichijo S, Imai Y and Itoh K (1999) Expression of the SART1 tumor rejection antigen in breast cancer. *Int J Cancer* **80**: 64–67

Kikuchi M, Nakao M, Inoue Y, Matsunaga K, Shichijo S, Yamana H and Itoh K (1999) Identification of a SART1-derived peptide capable of inducing HLA-A24-restricted and tumor-specific cytotoxic T lymphocytes. *Int J Cancer* **81** 459–466

Marchand M, van Baren N, Weynants P, Brichard V, Dreno B, Tessier MH, Rankin E, Parmiani G, Arienti F, Humblet Y, Bourlond A, Vanwijck R, Lienard D, Beauduin M, Dietrich PY, Russo V, Kerger J, Masucci G, Jager E, de Greve J, Atzpodien J, Brasseur F, Coulie PG, van der Bruggen P and Boon T (1999) Tumor regressions observed in patients with metastatic melanoma treated with an antigenic peptide encoded by gene MAGE-3 and presented by HLA-A1. *Int J Cancer* **80**: 219–230

Murayama K, Kogayashi T, Imaizumi T, Matsunaga K, Kuramoto T, Shigemori M, Shichijo S and Itoh K (2000) Expression of the SART3 tumor-rejection antigen in brain tumors and induction of cytotoxic T lymphocyres by its peptide. *J Immunotherapy* **23**(5): 511–518

Nestle FO, Alijagic S, Gilliet M, Sun Y, Grabbe S, Dummer R, Burg G and Schadendorf D (1998) Vaccination of melanoma patients with peptide- or tumor lysate-pulsed dendritic cells. *Nature Med* **4**: 328–332

Robbins PF, El-gamil M, Li YF, Kawakami Y, Loftus D, Appella E and Rosenberg SA (1996) A mutated β-catenin gene encodes a melanoma-specific antigen recognized by tumor infiltrating lymphocytes. *J Exp Med* **183**: 1185–1192

Robbins PF, El-gamil M, Li YF, Fitzgerald EB, Kawakami Y and Rosenberg SA (1997) The intronic region of an incompletely spliced gp 100 gene transcript encodes an epitope recognized by melanoma-reactive tumor-infiltrating lymphocytes. *J Immunol* **159**: 303–308

Rosenberg SA, Yang JC, Schwartzentruber DJ, Hwu P, Marincola FM, Topalian SL, Restifo NP, Dudley ME, Schwarz SL, Spiess PJ, Wunderlich JR, Parkhurst MR, Kawakami Y, Seipp CA, Einhorn JH and White DE (1998) Immunologic and therapeutic evaluation of a synthetic peptide vaccine for the treatment of patients with metastatic melanoma. *Nature Med* **4**: 321–327

Slamon DJ, Godolphin W, Jones LA, Holt JA, Wong SG, Keith DE, Levin WJ, Stuart SG, Udove J, Ullrich A and Press MF (1989) Studies of the HER-2/neu proto-oncogene in human breast and ovarian cancer. *Science* **244**: 707–712

Traverrsari C, Meazza R, Coppolecchia M, Basso S, Verrecchia A, van der Bruggen P, Ardizzoni A, Gsggero A and Ferrini S (1997) IFN-γ gene transfer restores HLA-class I expression and MAGE-3 antigen presentation to CTL in HLA-deficient small cell lung cancer. *Gene Therapy* **4**: 1029–1035

van der Bruggen P, Traversari C, Chomes P, Lurquin C, de Plaen E, van den Eynde B, Knuth A and Boon T (1991) A gene encoding an antigen recognized by cytolytic T lymphocytes on a human melanoma. *Science* **254**: 1643–1647

van der Bruggen P, Bastin J, Gajewski T, Coulie PG, Boul P, de Smet C, Traversari C, Townsent A and Boon T (1994) A peptide encoded by human gene MAGE-3 and presented by HLA-A2 induces cytolytic T lymphocytes that recognize tumor cells expressing MAGE-3. *Eur J Immunol* **24**: 3038–3043

Vasso A, Vaios K, John SH and Ian FCM (1997) Induction of HLA-A2-restricted CTLs to the mucin 1 human breast cancer antigen. *J Immunol* **159**: 5211–5218

Vincenzo R, Catia T, Alessandro V, Marccella M, Pier N and Claudio B (1995) Expression of the MAGE gene family and metastatic human breast cancer: implication for tumor antigen-specific immunotherapy. *Int. J. Cancer* **64**: 216–221

Yamauchi H, O'neill A, Gelman R, Carney W, Tenney DY, Hosch S and Hayes DF (1997) Prediction of response to antiestrogen therapy in advanced breast cancer patients by pretreatment circulating levels of extracellular domain of the HER-2/c-neu protein. *J Clin Oncol* **15**: 2518–2525

Yang D, Nakao M, Shichijo S, Sasatomi T, Takatu H, Matsumoto H, Mori K, Yamana H, Shirouzu K and Itoh K (1999) A gene coding for a protein possessing shared tumor epitopes capable of inducing cytotoxic T lymphocytes in cancer patients. *Cancer Res* **59**: 4056–4063

Zaks TZ, and Rosenberg SA (1998) Immunization with a peptide epitope (p369–377) from HER-2/neu leads to peptide-specificity cytotoxic T lymphocytes that fail to recognize HER-2/neu[+] tumors. *Cancer Res* **58**: 4902–4908

*© 2001 Cancer Research Campaign*
